# Heavy metals determination in honey samples using inductively coupled plasma-optical emission spectrometry

**DOI:** 10.1186/s40201-015-0189-8

**Published:** 2015-05-01

**Authors:** Hasan Mohammadi Aghamirlou, Monireh Khadem, Abdolrasoul Rahmani, Marzieh Sadeghian, Amir Hossein Mahvi, Arash Akbarzadeh, Shahrokh Nazmara

**Affiliations:** Department of Environmental Health Engineering, School of Public Health, Tehran University of Medical Sciences, Poursina St, Keshavarz Blvd, PO BOX: 6446-14155, Tehran, Iran; Department of Occupational Health Engineering, School of Public Health, Tehran University of Medical Sciences, Tehran, Iran; Department of Occupational HealthEngineering, School of Health and Nutrition, Shiraz University ofMedical Sciences, Shiraz, Iran; Department of Occupational Health Engineering, School of Public Health, International Campus, Shahid Beheshti University of Medical Sciences, Tehran, Iran; Center for Solid Waste Research, Institute for Environmental Research, Tehran University of Medical Sciences, Tehran, Iran; Department of Epidemiology and Biostatistics, School of Public Health, Tehran University of Medical Sciences, Tehran, Iran

**Keywords:** Honey, Inductively coupled plasma-optic emission spectrophotometry, Heavy metals

## Abstract

**Background:**

Honey contains a complex mixture of carbohydrates and other minor substances. Elements are minor constituents of honey that may threaten the human health in excess concentrations. So, determining the metals in honey helps its quality control as a food product. The aim of this study was to determine the concentrations of some metals in Iranian honey.

**Methods:**

This study was performed in four regions of Ardabil, a province of Iran. Honey samples (n = 25) were digested in microwave oven by nitric acid and hydrogen peroxide, then analyzed using inductively coupled plasma– optic emission spectrophotometry (ICP-OES).

**Results:**

No significant differences were observed in cadmium, zinc, nickel, and chromium levels between regions (P > 0.05). Zinc was the most abundant metal in honey samples (1481.64 μg/kg). Some metals had higher concentrations in the East region because of existence more industries there. The highest mean of lead level was 935.48 μg/kg in the East and the lowest was 205.4 μg/kg in the South region. The concentrations of metals were compared with recommended limits for foods. Some of them were higher than standard levels (lead) and some were lower than those (cadmium).

**Conclusions:**

Metals are released into the environment through their use in industrial processes and enter the food chain from uptake by plants from contaminated soil or water. Metals concentration in various places depends on many variables, leading to their different concentrations in honey. Some control measures like the quality control of food products, monitoring the soil in agricultural regions and limiting the use of fertilizers are recommended.

## Background

Honey, produced by the honeybee, is a natural supersaturated sugar solution, which has been consumed as a high nutritive value food and is composed of a complex mixture of carbohydrates [1]. This natural product is so valuable as the only concentrated form of sugar available worldwide [2] and is also used as a food preservative. It also contains the certain minor constituents like enzymes (glucose oxidase, catalase, phosphatases), glucose and sucrose (65–75% of total soluble solids), proteins, amino and organic acids, vitamins, lipids, volatile chemicals, flavonoids, phenolic acids, and minerals [3,4]. The biochemical properties of honey and its quality are related to honey maturity, climatic conditions, production methods, processing and storage conditions, as well as the nectar source of the honey [5-9].

**Figure 1:**
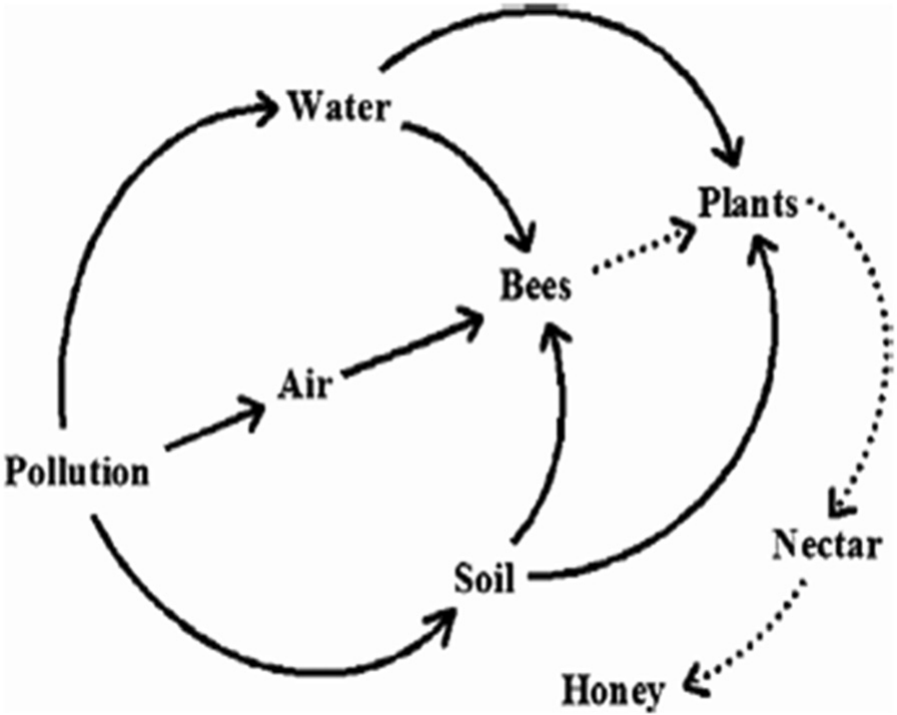
Natural and anthropogenic sources of metals in honey [33].

Elements are minor constituents of honey. The kind of these elements in honey is related to the type of raw floral materials, i.e., the nectar, the pollen, and the honey dew, which are collected by bees [10-13]. Metal concentrations in different honey types depend largely on the elemental composition of flowers, with regard to their botanical and geographical origin [14,15]. These metals may come from external sources such as industrial smelter pollution, industrial unit emissions, and improper procedures during honey processing and maintenance stages. Also, the origin of metals in honey can be agrochemicals such as organic mercury, cadmium-containing fertilizers and arsenic-based pesticides (Figure 1) [14-16].

The presence of metals in honey may threaten the health of human as a consumer [17]. These metals can damage the quality of human life when they accumulate to a toxic concentration level [18]. In recent years, the concentrations of different metals in honey have been determined in some countries, such as China [17], Italy [14], France [19], Croatia [4], Slovenia [20], Poland [21], and Turkey [22-25]. Also, heavy metals in bees and in bee products have been the subject of many other various studies [26-32].

Heavy metals pollution is a serious problem in Iran because of the mining, smelting, and metal treatment industries. Heavy metals pollution affects the quality of productions, as well as the qualities of the atmosphere and waters, threatening the health and life of human beings and animals via the food chain. Although in Iran honey is produced and consumed on a large scale, there is a lack of information to determine the heavy metals in Iranian honeys [17]. In addition to its environmental importance, determining the heavy metals is important for the quality control of honey as one of the most complex food products. Therefore, the objective of the current study was to determine the concentrations of some heavy metals like copper (Cu), zinc (Zn), cadmium (Cd), lead (Pb), arsenic (As), nickel (Ni) and chromium (Cr) in Iranian honey. The results of such studies can help prevent the mentioned problems and improve the healthy honey consumption. These results can lead to considering the origins of honey contaminants such as soil type and air pollution, regarding the food safety in health policy, and providing best quality of food will protect public health and preserve consumer confidence.

## Methods

### Sample collection

**Figure 2:**
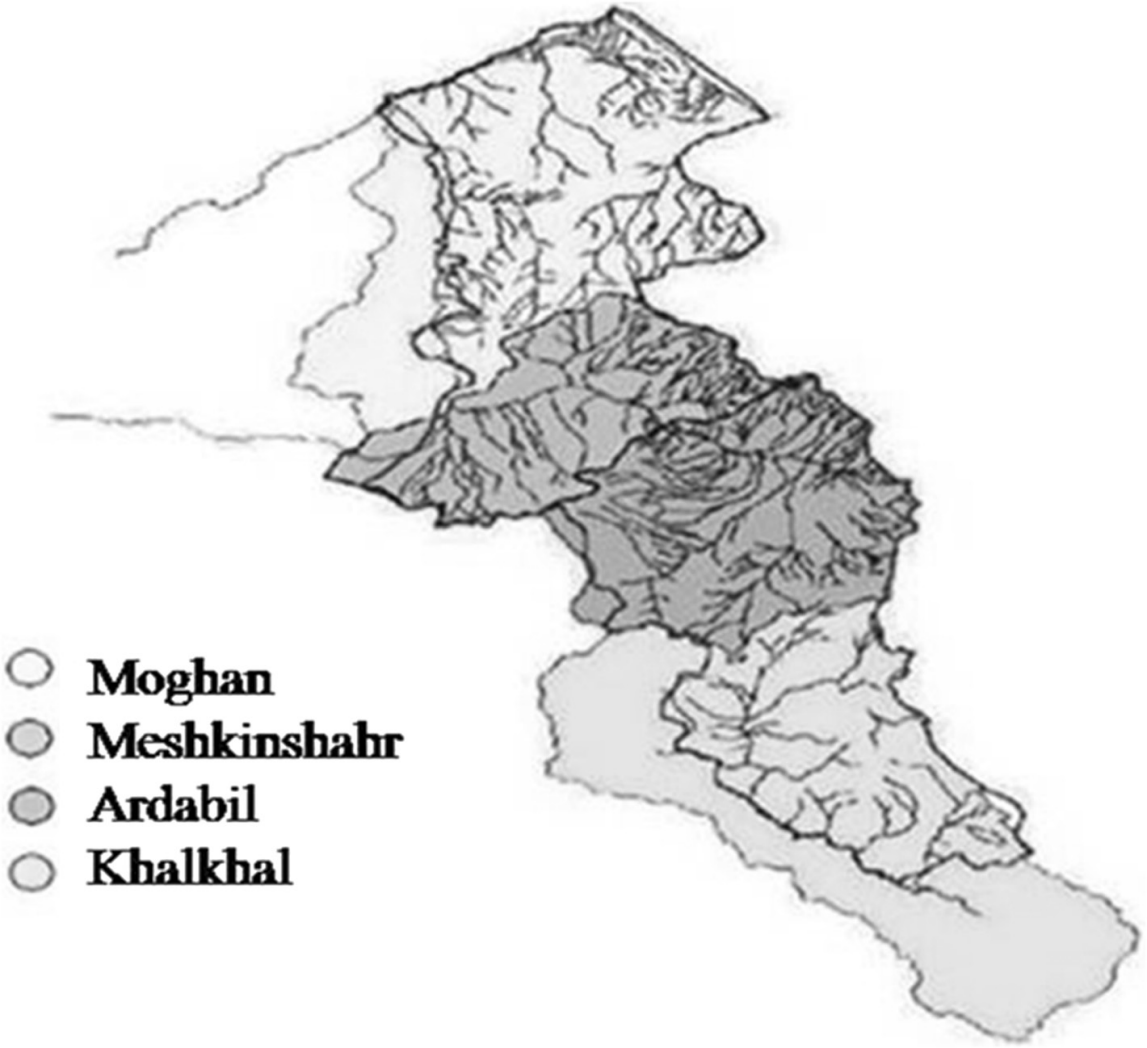
Geographical location of the four studied regions in the Ardabil province.

This cross sectional study was performed in Ardabil, a province in North West of Iran, in 2013. During these year, a total of 25 samples of multi floral honey were collected from individual beekeepers in four regions of Ardabil: East (Ardabil County, n = 6), North (Moghan County, n = 7), South (Khalkhal County, n = 6) and West (Meshkinshar County, n = 6) (Figure 2). The Eastern region is the most populated, urbanized and industrialized in comparison with the other regions, particularly the South and North.

All honey samples (400 g) were provided by the local association of bee keepers with guaranteed origin and made by traditional procedures in the honey-producing region; all samples were collected in clean and closed glass jars and immediately transferred to the laboratory of Department of Environmental Health at Tehran University of Medical Sciences; all samples were stored in glass bottles and kept at 4–8 ºC in dark place until analysis.

### Apparatus

Determination of Heavy metals was performed using an Octople Reaction System (ORS) inductively coupled plasma– optic emission spectrophotometry (ICP-OES), Spectro Arcos OES EOP (Germany).

Table 1 shows the ICP-OES operating conditions to do all metal analyses. A microwave oven (MARS 5, CEM) was used to digest the samples and their pretreatment. Instrumental parameters and settings for microwave digestion of samples were 15 min/600 W at 120°C, 20 min/600 W at 180°C and venting for 20 min.

**Table 1:** **Instrumental characteristics and settings for ICP-OES**

**Spectrometer**	**Agilent 7500ce with ORS**
**Nebulizer**	Micromist
**Interface**	Interface
**RF generator (W)**	1550
**Argon flow rate (L min** ^**-1**^ **)**	0.85
**Nebulizer pump (rps)**	0.10
**Scanning condition**	Number of replicate 5, dwelling time 1 s
**Scanning mode**	Pulse
**Reduction gas flow (L min** ^**-1**^ **):**	
**H**_**2**_	3.5
**He**	4.0
**Internal standard**	^45^Sc, ^89^Y, ^159^ Tb

### Reagents and chemicals

All reagents were of analytical grade unless otherwise stated. Double-deionized water produced by Milli Q water purification system (Millipore) was used in all dilutions. The stock solutions of Cu, Cr, Cd, Pb, As, Ni and Zn (1000 mg/L, ICP standard CertiPUR) were purchased from Merck (Germany) and the elements standard solutions were prepared by diluting them. The same procedure was applied to prepare a solution of ^45^Sc, ^89^Y, ^159^ Tb as an internal standard in the ICP-OES technique. Using the internal standard is recommended in routine analysis by ICP-OES to compensate the possible drift during long term runs and correct the matrix effects. Honey samples were digested by concentrated nitric acid (65% HNO_3_ suprapure, Merck, Germany) and hydrogen peroxide (30% H_2_O_2_ pure p.a, Chempur, Poland).

### Analytical procedures

To determine seven mentioned metals, all honey samples were prepared according to the following procedure: exactly 1 gram of each sample was weighed in PTFE vessels and dissolved in 10 milliliter concentrated nitric acid (HNO_3_). After that, samples were digested in the microwave oven [25]. This process was set in a closed system, so the sample decomposition had no contact with external surroundings, thereby reducing the risk of contamination. Blank solutions were prepared by nitric acid.

ICP-OES technique is able to do the multi elemental analysis with excellent sensitivity and high sample throughout, resulting in high precision and accuracy. So it was used to determine the interested heavy metals in honey samples similar to other studies [14]. In some studies the analysis and measurements of heavy metals are done by atomic absorption and emission spectrometries [33].

The precision of the analytical method was evaluated in terms of repeatability of the experimental results of real samples and expressed as standard deviation (S.D). The accuracy was verified by calibration (using standard solutions). Additionally the internal standard (^45^Sc, ^89^Y, ^159^ Tb) was applied for ICP-OES technique to correct the matrix effects.

### Data analysis

The statistical calculations and analysis were performed using SPSS version 18 (SPSS Inc., Chicago, IL, USA). Some tests such as one-way ANOVA and *T*-test were used for data analyzing. The level of significance was taken as p < 0.05.

## Results

To ensure the reliability of the results, the analysis of recovery rate was carried out by spiked honey samples for Cu, Cr, Cd, Pb, As, Ni and Zn. There was a good accuracy with recovery rates of 95–100% for metals (Table 2). As regards honey is mainly contains mineral trace elements, such as calcium, copper, iron, magnesium, manganese, potassium, and other minerals, in this study it was considered by applying the blank samples to obtain the accurate data. Blank honey samples are identified by the absence of compounds of interest (heavy metals), with prior injection into the detection system.

**Table 2:** **Heavy metals concentration and recoveries in spike honey samples**

**Element**	**Certified value (μgg** ^**−1**^ **)**	**Measured value (μgg** ^**−1**^ **)**	**Recovery (%)**
**As**	5.67	4.68 ± 0.30	95
**Cd**	0.013	0.013 ± 0.001	100
**Cu**	5.64	5.60 ± 0.20	99
**Pb**	0.47	0.45 ± 0.03	97.2
**Zn**	12.5	10.9 ± 0.9	88.4
**Cr**	0.3	0.29 ± 0.03	99
**Ni**	0.91	0.87 ± 0.04	96

Table 3 indicates the concentration of metals in honey samples. The basic statistical data such as the number of samples, mean values, minimum and maximum values can be seen. Zinc is the most abundant metal in all honey samples having an average of 1481.64 μgkg^−1^ (ranged from 122.86 to 6638.55 μgkg^−1^). The other major metals, i.e. Cu, Cr, Cd, Pb and Ni have the considerable lower averages in comparison with zinc. In the present study, the highest cadmium level was in the East region and the lowest was in the North. There were no significant differences between cadmium levels in various regions (P = 0.107). Also, no significant differences were observed in zinc, nickel, and chromium levels between regions (P > 0.05).

**Table 3:** **Concentrations of heavy metals in different types of honey (μgkg** ^**−1**^ **)**

**Region**	**N**	**Analyzed Metals (μgkg** ^**−1**^ **)**							
		**Statistics**	**As**	**Cd**	**Cr**	**Pb**	**Ni**	**Zn**	**Cu**
**North**	7	mean	<11.87	17.58	820.74	324.75	608.17	775.74	94.74
		Minimum	<11.87	1.36	172.37	144.95	65.04	122.86	27.65
		Maximum	<11.87	82.74	1220.3	960.88	1094.49	2265.38	150.12
		SD		28.99	316.24	285.02	301.38	746.78	49.1
**South**	6	mean	<11.87	12.84	887.52	205.4	630.07	2725.09	97.96
		Minimum	<11.87	7.49	850.39	117.46	585.07	433.84	71.43
		Maximum	<11.87	19.12	932.38	380.17	677.77	5491.75	143.71
		SD		4.96	33.92	93.3	34.61	1888.51	25.84
**East**	6	mean	<11.87	53.64	947.24	935.48	707.24	1043.47	212.53
		Minimum	<11.87	11.48	895.71	431.64	622.68	240.45	96.75
		Maximum	<11.87	125.88	1020.67	1627.82	805.66	2031.42	631.79
		SD		47.97	52.92	473.51	77.82	753.56	211.34
**West**	6	mean	<11.87	28.08	956.64	595.16	593.92	1499.92	591.49
		Minimum	<11.87	9.16	711.58	134.28	450.96	169.30	85.48
		Maximum	<11.87	64.92	1165.68	871.24	879.38	6638.55	2872.74
		SD		19.14	151.95	246.1	143.6	2543.95	1118.22
**All samples**	25	mean	<11.87	27.62	899.75	507.58	651.78	1481.64	243
		Minimum	<11.87	1.36	172.37	117.46	65.04	122.86	27.65
		Maximum	<11.87	125.88	1220.3	1627.82	1094.49	6638.55	2872.74
		SD		32	184.03	402.14	173.29	1709.81	559.3

Statistical analysis by ANOVA showed a significant difference between lead levels (P = 0.002) for honey samples in various regions. Furthermore, the highest mean of lead level was 935.48 μgkg^−1^ in the East and the lowest was 205.4 μgkg^−1^ in the South region. The highest and lowest levels of copper were seen in the West and North, respectively. No significant differences were observed in copper levels between regions (P = 0.374).

## Discussion

The aim of this study was to determine the concentrations of heavy metals in honey. It is worth to mention that there are no documented studies indicating the rate and pattern of metals in honey in Iran.

Based on our findings, the lowest and highest mean copper concentrations in the honey samples were in the North (94.74 μgkg^−1^) and West (591.49 μgkg^−1^), respectively. The mean of copper content in honey samples from all four regions was 243 μgkg^−1^. The provisional tolerable daily intake (PTDI) for copper, set as a limit for metal intake based on body weight for an average adult (60 kg body weight) is 3 mg [34]. Copper is a vital element to the health of all living things and in humans. However, too much ingestion of copper can lead to adverse health effects in the body. So, it is necessary to consider the daily intake of copper from different sources like food. In present study, the mean level of copper was much lower than those reported in previous surveys in, Italy (647,310 and 890 μgkg − 1) [14,35,36], Ireland (0.2 mg 100 g^−1^) [37], and New Zealand (0.25 mgkg^−1^) [38], but higher than in other studies in the Black Sea Region of Turkey (9.75–35.8 μgkg^−1^ [23], china (33.98 μgkg^−1^) [17] and New Zealand (163–182 μgkg^−1^) [39]. These are not completely consistent with our findings that may be due to differences in the studied regions like using different fertilizers or the diversity in practice of growing the plants.

Based on our findings, cadmium concentrations ranged from 1.36 to 125.88 μgkg^−1^with a mean value of 27.62 μgkg^−1^ that was under the maximum permissible concentration (200 μgkg^−1^) of cadmium [40]. Cadmium concentrations in this study were lower than those reported in Italy (305 μgkg^−1^) [39], but higher than in other studies in china (1.34 μgkg^−1^) [17], Turkey (0.9–17.9 μgkg^−1^) [24], Macedonia (3.63 μgkg^−1^) [41], Poland (0.015 mgkg^−1^) [21], Italy (3.91 μgkg^−1^) [14], Romania (0.015 μgkg^−1^) [42], Turkish (0.32 μgkg^−1^) [43] and Turkey (0.38–2.03 μgkg^−1^ [23]. This is not completely consistent with our findings may be due to differences in the studied regions in various surveys. Cadmium is released into the environment through its use in various industrial processes, and enters the food chain from uptake by plants from contaminated soil or water. Therefore, the cadmium concentration in various places depends on many variables, leading to its different concentration in honey samples in those places.

Based on our findings, the lowest and the highest mean of lead concentrations were 205.4 μgkg^−1^in the honey sample from the South and 935.48 μgkg^−1^ in the East. The mean of lead content in honey samples from all four regions was 507.58 μgkg^−1^ that last two concentrations exceed the standard level of 300 μgkg^−1^, recommended by FAO/WHO/1984 [40]. Lead can be found in many products and locations. Lead gets into the air and then mixes with the soil near one of its sources, entering into the plants. So, lead concentration in some food like honey can be elevated depending on a lot of variables. In this study, lead concentration in honey samples from the East is higher than other regions. Therefore the soil contamination with lead may be occurred in the East, causing its uptake into the plants feeding bees. Also, lead has no beneficial role in human metabolism and can cause some health disorders. Thus, it must be considered seriously.

Antonescu and Mateescu [44] reported that no samples of Rumanian honey contained Cd and As, while it contained lead of 0.1–200 μgkg^-1^ in the range within the limits imposed by the last regulations of the Codex Alimentarius [45]. The measured lead concentration was almost higher than those found in honey samples from china (33.98 μgkg^−1^) [17], Croatia (65.2 μgkg^−1^) [4], New Zealand (0.017 mgkg^−1^) [38], Turkey (17.6–32.1 μgkg^−1^) [25], Poland (0.048 mgkg^−1^) [21], Romania (0.07 μgkg^−1^) [42] and in samples from Taiwan and mainland China (0.007–0.029 mgkg^−1^) [46]. However, the lead concentrations found in this study were lower than those found in honeys from Italian areas (2370 μgkg^−1^) [47], Slovenia (5.94 mgkg^−1^) [20] and Italy (620 μgkg^−1^) [39].

The mean arsenic concentration in all honey samples was <11.87 μgkg^−1^, which was below the maximum allowable level (10-500 μgkg^−1^), regulations of the Codex Alimentarius [45] and Commission Regulation [48]. In comparison with the very few measured contents in the literature, the arsenic levels obtained were higher than levels found in Siena County, Italy (6.59 and <0.5 μgkg^−1^) [14,35]. However, the arsenic concentrations were lower than those found in honeys from Croatia (19.7 μgkg^−1^) [4], Slovenia (1.24–1.49 mgkg^−1^) [20], and New Zealand (0.08 mgkg^−1^) [38]. Environmental pollution factors that may contribute to the presence of arsenic in honey may be caused by non-ferrous metallurgy, factories, and agrochemicals such as fertilizers, and arsenic-based pesticides. Arsenic is also found in food, water, soil, and air. It is absorbed by all plants, therefore can present in honey. Some control measures such as the quality control of food products and limiting the use of arsenic-based pesticides are recommended.

Zinc can be toxic at high concentrations and plants affected may show symptoms similar to those found in other heavy metal toxicities [49]. In most cases, excess Zn generates reactive oxygen species and/or displaces other metals from active sites in proteins [50]. Zinc is an essential mineral required by the body for keeping a healthy immune system, building proteins and other processes. The most important sources of anthropogenic zinc in soil come from discharges of smelter slags and wastes, mine tailings and the use of commercial products such as fertilizers and wood preservatives that contain zinc. The average recommended as daily intake in foods is estimated to be 12–15 mg/day for zinc [51-53]. In the present study, the maximum and minimum Zn concentrations were 6638.55 μgkg^−1^ in the West and 122.86 μgkg^−1^ the North. The results showed that Zn was the most abundant metal in honeys with an average value of 1481.64 μgkg^−1^. This average Zn concentration was lower than those found in honey samples from Siena County of Italy (1820 μgkg − 1) [14], Italy (3205 μgkg − 1) [39], and Slovenia (3.61 mgkg-1) [20]. However, the obtained Zn levels were higher than levels found in china (1329.5 μgkg − 1) [17], New Zealand (1.18 mgkg-1) [37], Taiwan and mainland China (0.996 mgkg-1) [54]. Keeping honey in galvanized containers might be the source of Zn contamination in honeys [55]. Some researchers have expressed that diverse metal concentrations in honeys is extremely reliant on the kind of flowers utilized by bees and it can be the chief source of Zn contamination [15]. Although zinc is an essential element for human body, high intake of it may be led to adverse health effects. Thus, the quality control of food products is necessary.

Based on our findings, the mean of nickel content in honey samples from all four regions were 651.78 μgkg^−1^. The mean of nickel in the East region is more than other ones that may be related to more industrial sources of nickel there. The intake of nickel via food is related to several factors such as the source of nickel and distance from the contamination source. Nickel is present in the air, water, and soil and is generally distributed uniformly through the soil profile. The level of 5 mg/kg body weight/day was determined for nickel by joint FAO/WHO Expert Committee on Food Additives (JEFCA). Our findings were not similar with the previous reports in which nickel concentration was not detected [43,56,57].

Chromium (Cr) is listed by the Environmental Protection Agency as one of the 129 priority pollutants and one of the 14 most noxious heavy metals [58]. The general population is exposed most often by ingestion through chromium content in soil, food, and water [59]. Trivalent chromium is the most common natural state of chromium and an essential nutrient. Its Recommended Daily Intake is 30 to 100μg/day for adults. However, the primary route of non occupational exposure to chromium is food ingestion. Chromium in foodstuffs is considered to be in the trivalent form [60]. Based on our findings, chromium concentrations ranged from 172.37 to 1220.3 μgkg − 1 with a mean value of 899.75 μgkg − 1. The highest mean chromium concentrations were 1220.3 μgkg − 1 in the honey samples from the North region. According to the Expert Group on Vitamins and Minerals (EVM) a total daily intake of about 0.15 mg chromium (III)/kg bw/day (or 10 mg/person/day) would be expected to be without adverse health effects, whereas the WHO considered that supplementation of chromium should not exceed 250 μg/day (European Food Safety Authority 2010) [61]. According these regulations and data, chromium concentrations in studied honey samples can be considered in an acceptable range. Because of the lack of studies in this field, it is not possible the comparison of present study with other investigations. Finally, the mean concentrations of each heavy metal were compared between four regions. There were significant differences (P < 0.05) between regions for all heavy metals excepting arsenic.

Many people would like to drink their tea with honey. Since, black tea has heavy metals, the overall heavy metal intakes should be considered [62,63].

## Conclusion

The present study indicated all types of honey contain metals and the metals concentrations vary among different regions because of some variables. Findings showed that zinc and arsenic had the maximum and minimum concentrations, respectively. Some geological and geochemical parameters may affect the honey chemistry. The proximity to the industries, having different types of soil, using various fertilizers, and the diversity in practice of growing the plants may be led to some differences between regions. However, further studies of the honeys are required, especially respecting the comparison of metals concentration with standard level based on body weight and honey consumption. In this study, it was difficult to compare the obtained results with related standards, because some standards were based on daily or weekly intake of metals, while there was no data about daily consumption of honey by peoples. Finally, according to literatures, some recommendations can be mentioned, such as limiting the consumption of hazardous fertilizers, monitoring the soil in agricultural regions, considering the distance of agricultural lands and flower gardens with industries, controlling the quality of food products, providing some accurate standards limits for hazardous compounds in the foods, and monitoring the waters that is used for agriculture and flower working. Since, due to the corrosive effect of honey, its contact with stainless steel surfaces and galvanized containers can be a source of chromium and zinc, using proper containers for storage of the honey is recommended.
